# Online sports fans communities: humor, trivial knowledge, and anti-modern tendencies

**DOI:** 10.3389/fspor.2023.1280519

**Published:** 2023-10-11

**Authors:** Orr Levental

**Affiliations:** Department of Phsyical Education, Tel-Hai Academic College, Qiryat Shemona, Israel

**Keywords:** Israel, sports fandom, fans, online communities, humor, memes

## Abstract

Recent technical developments and the widespread use of social networks have led to the emergence of a variety of online communities built around common interests. Among these virtual communities, one notable category revolves around sports fans. This conceptual article uses several examples of online sports fan communities in Israel to analyze their core elements. Within this context, the article focuses on the significance of humor, trivial knowledge, and counter-modern concepts as the key elements fostering unity among fans. Humor serves as a common thread that encourages interactions, also providing a platform for fans to showcase their niche knowledge, symbolizing their dedication to fan culture. Meanwhile, the prevalence of counter-modern inclinations within these communities highlights the fans' constant devotion to the sport, demonstrating a profound loyalty. Collectively, these elements signify a shift from supporting specific teams to becoming keen fans of the broader sporting domain.

## Introduction

Early in the 2009–2010 football season, Avi Luzon, the chairman of the Israel Football Association, said that, from his perspective, the quality of play in the Israeli league ranks sixth in all of Europe. As soon as this statement was released, criticism in the media and public sphere focused on Luzon's overblown adulation of Israeli football. Since then, this phrase has served as a metaphor for Israeli football fans' self-criticism and acceptance of the slightly subpar quality of Israeli sports compared to that of other nations. Many fans, however, do not bemoan this situation but rather accept it as a particular quality or, at the very least, do not view it as a hindrance to their enjoyment and fandom.

Fans naturally support a particular team and anticipate continual success from them. As a result, there is rivalry amongst supporters of various teams. But new fan communities have been formed in recent years, especially in light of online social networks. These do not cater to supporters of any particular club but rather to all Israeli football fans as a whole. These communities, covered in the current article, have created a new element of fan culture in place of the club's success. It encourages the idea of common interest and romanticizes sports' simplicity. In the context of Israeli football, this trend has led to the popularization of several online communities that allow any Hebrew-speaking football fan to participate. Among the most popular communities are *Kaduregel Shefel* (Low football, 25K followers, 10 posts per week on average)*, Tsiyun 3* (3/10, 41K followers, 5 posts per week on average)*, Kadureglanim sh'hunatiim and svirim* (reasonable and down-to-earth footballers, 71K followers, 10 posts per week on average)*, memem of kaduregel* (Football memes, 24K followers, 5 posts per week on average), and *Kaduregel Shefel Miluim* (Low football reserves, 6K followers, 20 posts per week on average)*.* By considering three topics—humor as a point of commonality, the particular importance of marginal knowledge, and anti-modern tendencies—the current article aims to analyze the significance and characteristics of these online groups. To achieve this objective, approximately 100 popular posts from each group were incorporated, from the group's inception to August 2023. While this article draws its foundational concepts from Israeli online communities, it endeavors to analyze a sports fandom phenomenon that transcends geographic boundaries. Emphasizing the local cultural context enhances understanding of the specialized and cultural elements that reside within the broader framework.

## Humor as a common ground

In his book “*The Ecstasy of Communication*”, (1) argues that the modern world is characterized by a flow of recurring ideas and symbolic information through various communication outlets. One notable illustration of this idea is the relatively recent popularity of internet memes. Memes constitute a way of exchanging elements of cultural and social thoughts, which are virally communicated on various media platforms and channels (2). This phenomenon also relates to Baudrillard's *Simculacra,* which he defined as a reflection of real objects that generate, replicate, and exist as part of hyperreality (3). Memes, therefore, do not focus on the original context or meaning. However, they show the ability of various, usually anonymous, people to exhibit creativity in using the same template with slight alternations to create new, relatable shared content. Thus, social network sites are the medium; memes contain symbols, semiotics, and trivial knowledge; and humor is the common denominator on which this form of communication thrives.

Veatch (4) describes humor as the existence of two elements simultaneously: “The perceiver has in mind a predominating view of the situation as being normal” (164); “The perceiver has in mind a view of the situation as constituting a violation of a subjective moral principle that is, some affective commitment of the perceiver to the way something in the situation ought to be is violated” (163). According to Veatch, humor is based on a circumstance everyone can relate to, while presenting an understandable change or twist. In the context of memes, (5) notes that memes as a humorous tool require users' literacy of the subject and to be broadly accessible to diverse identities and perspectives. That is, a meme can serve as a point of connection for people from a wide range of backgrounds as long as it is relatable and understood. In the case of Israeli football, fans of different teams, socioeconomic statuses and places of living are brought together by their knowledge of local football.

*Hutza Me'ekshero* (Taken out of Context), a book by (6), was published in Israel in 2001. The book included a collection of absurd quotations from football coaches, players, and journalists. The book became a success, leading to the publication of four other similar books. One recurring figure in the books was Israeli striker Alon Mizrahi. Among his quotes were: “*I have nothing to prove, and I proved it*” and “*I would like to play in Spain or Europe*”. Fans in Israel unjustly viewed Mizrahi as a representation of an unintelligent footballer. As a result, his name frequently appeared in jokes, and his now-famous quotes were repeated and paraphrased. Even outside the Israeli football sphere, Mizrahi was a relatable and well-understood “meme” in those early internet days. And supporters of all teams, including his own, got in on the joke and spread it throughout various platforms. This was the first significant instance of sports-related content providing humor-based interpersonal communication. It used humor and general knowledge of Israeli football figures and events as a connecting tool. The technological advancements of the internet, especially the rise of social media, further developed this phenomenon. By increasing the number of possible participants, the necessary knowledge to participate in the humorous exchange became more trivial and anecdotal.

According to Broughton (7), social media can fuel rivalries between fans of different teams. This is also apparent in the Israeli context, in which football fans maintain violent online discourse (8). The reason is that sports fandom requires dichotomic affiliations, and each team has its colors, history, symbols, players, and staff (9). Fans will show appreciation toward a player and defend his actions because he plays for their team and therefore is considered one of them. Alternatively, when the subject of a meme is not focusing on the quality of a specific team or a player, it does not incite conflict. Instead, it allows fans to come together to celebrate broader shared interests. Moreover, the common ground on which this humor thrives, as discussed in the following sections, is the trivial knowledge and the anti-modern tendencies that often include the perceived mediocracy of Israeli football. Martin and Ford (10) argue that humor has several social functions for individuals and groups. One of them is releasing prejudice and fostering social cohesion in intergroup relations. Due to the inherent competitiveness in sports, humor can be a powerful common ground on which different and even rival groups can cooperate. Humor-based communities and pages on social media dissolve the exclusivity between a fan and his favorite team and make him a fan of the sport in general. By creating, sharing, and liking Israeli football memes, one is not obliged to identify himself as a fan of a specific team but as a member of a larger social circle, including all football fans as a whole.

## Trivial knowledge as an indicator of fandom's quality

According to (11), complex jokes, to a certain extent, are perceived as funnier. Thus, referencing more trivial information in memes makes the readers perceive their quality as higher. The specificity of the football memes shifted the emphasis from well-known information to information that is only known to those with a thorough understanding of the sport. Moreover, knowledge is an essential part of the fans' identity. Sports fans, as well as in other fields, take pride in their devotion. Traditionally, hardcore fans were measured by their attendance in the stands, and those who followed the team on home and away games were considered the most loyal (12). However, given current tendencies in the globalization of sports, particularly football (13), some fans choose alternative ways of demonstrating their dedication (14). While some fans continue to support their team in the traditional way of attending matches, others focus on the consumer aspect of fandom (15). In either case, fans' knowledge about the club's history, players, results, and current state of affairs is still highly valued (16). A high level of knowledge demonstrates a person's passion for the subject and the time he invests in his devotion. It is not exclusive to football fans or sports fans in general, but it is a universal indicator of dedication to a specific issue or field.

The rise in popularity of fantasy sports serves as the best illustration of the importance of knowledge and understanding in sports. At its core, fantasy sport is a game designed to test the participants' understanding of real-life performance. The most crucial element is a thorough knowledge of players’ fitness, form, and abilities; teams’ strategies, schedules, and momentum; and awareness of important statistics, rules, weather, etc. These, in addition to long-term planning and consistently good decision-making (17). According to (18), not only are good fantasy players experts in sports and use their knowledge for success in the game but the audience for displaying their knowledge-derived expertise in fantasy sports is made up of friends and competitors. Therefore, demonstrating deep knowledge of sports strengthens the self-perception and the perception of others about one's fandom.

Like fantasy sports, being part of the football meme culture requires familiarity with the best and less-known players. Knowing Messi or Ronaldo is banal, while being aware of a backup player in some mid-table team reflects depth. Furthermore, because everyone wishes to exhibit this depth, memes about the leading players in Israeli football seem much less popular than those that refer to retired players or those who play for low-ranking teams. The posts that create the most traffic on the humorous sports pages on Facebook usually involve name-dropping or mentioning semi-esoteric events. Thus, Liking or sharing a post about some player who retired two decades ago signals that one has understood the reference or allusion. The age range of the pages' members can also be inferred from the display of trivial knowledge. A sports fan born in the early 1980s might be familiar with the names of well-known football players like Lev Yashin or Just Fountain, but may not be familiar with their appearances. Thus, in 2023, referencing Israeli footballers from the 1990s might appeal to people 35 years of age and older. This target audience will recognize the name of the Israeli international footballer Pini Balily and be aware of his unique facial feature of thick eyebrows. Therefore, referencing his eyebrows allows users to exhibit knowledge by signaling that they understand this jest. Popularizing specific content contributes to defining the subculture of the online community and its demographic while helping create group cohesion based on semi-obscure knowledge as a symbol of value.

Memes are a widespread worldwide phenomenon and there are numerous groups on all social media sites, including Reddit, X, Instagram, and others, devoted to this kind of humor. On most websites, you may also find communities related to specific subjects like TV shows, music, various sports, and sports clubs. For instance, the “Football memes” group, which is active on Facebook and X, has more than a million subscribers. However, the characteristics reviewed so far, humor and trivial knowledge, are largely culture-dependent. The group begins to lose its local identity and communal traits as it becomes more appealing to larger audiences. Consequently, a niche group will be seen as having more prestigious qualities and possessing more communal traits.

## Anti-modern tendencies and embracing mediocrity

Nostalgia is an integral part of sports fandom. It affects stadium architecture (19) and the design of uniforms (20) and shapes tourism motives and trends (21). Nostalgia is the intangible expression of sports heritage and is often linked with a subjective perception of simpler times and less alienation. Sports, in the eyes of the nostalgic fan, is not about money or worldwide fame but rather about emotions, pride, and local communal aspects (22). Nevertheless, the sports fans on the humorous sports pages on Facebook are not blinded by nostalgia. They understand that football today is better regarding the quality of play or the convenience of watching matches on television or in the stands. However, reminiscing and celebrating earlier sports has social advantages. Batcho (23) argues that nostalgia proclivity was associated with emotional and instrumental social coping and goal-directed methods such as planning, action, and positive reframing. Nostalgia can boost self-esteem, provide comfort and solace, and improve psychological well-being (24). Sports nostalgia allows a fan to maintain his apparent “pure” dedication to the sport. While sport is a multifaceted industry, reminiscing about the past buffers individuals from being linked with the contemporary characteristics of sports, such as mediatization, globalization, consumerism, commercialism, and even professionalism. Nonetheless, the nostalgic fan still proudly upholds archaic properties of sports, such as passion, devotion, and loyalty, all through a romantic prism.

The *Kaduregel Shefel* (low football) project, which has accounts on all major social media platforms, is one noteworthy example of this trend. This project, which now includes an online merchandise shop, tours, galleries, seminars, and more, is described by its creators as a cultural experience in Israeli and international low football leagues. It highlights football's simplicity, untainted by sports' modernity. It accomplishes this by focusing on two areas of football: traditional and esoteric. The first is football from the 1990s or 2000s, which is no longer relevant in today's sports. Aside from focusing on retiring players, it also showcases the aesthetics of stadiums, balls, mascots, magazines, and clothing design from that era. They do this by appealing to fans' nostalgic instincts. Nonetheless, the vital feature of the content is the humorous nature of outdated sports components. While recalling a great player may be nostalgic, displaying a club uniform and focusing on a sponsor no longer in business aims to demonstrate the absurd spectrum of fandom between sports laden with personal and cultural meaning and sport that is temporal and only relevant for a specific place and time. As I previously stated, it is comical since it is relatively obscure and requires anecdotal knowledge held by only a select demographic of football fans.

The second area of this project is esoteric football in Israel and around the world. The community's members share stories and images of stadiums, players, and spectators from lower-tier leagues or regional competitions. Similar to other social media accounts like *varzeiroocial* on Instagram and *out of context* football onX, they touch on the minuscule incidents of the sport, snickering at those participating in it, and ridiculing the extreme seriousness with which some people consider it. This popular pattern stems from supporters' desire to undermine the sport's perceived strive for perfection. To demonstrate that people appreciate sports even if they are neither professional athletes nor supporters of unsuccessful lower-league teams. Adopting such an outlook provides a comparison between a supporter of an elite team in a top-tier league and one of an unpopular mid-table club in a local league regarding dedication to the sport and depth of understanding of it.

This is made possible by accepting mediocrity as a natural feature rather than something to be actively avoided. Recognizing mediocrity allows the fan to lessen expectations, put things in perspective, and turn flaws into an ordinary aspect of sports fandom. Thus, by employing humor, demonstrating knowledge, and eschewing professionalism, the fan is no longer required to defend his club's performance; instead, he can engage in an inclusive community of sports fans.

## From a supporter to a fan of the sports

After decades of research, the scholarly literature recommends numerous models for categorizing sports fans based on their motivations, behaviors, and interests (15, 25, 26). They all, however, refer to sports fans who follow and support a certain team. Even when there is a low sense of attachment, as in the case of new fans who follow a club's recent success or because of an interest in a particular player, “the sports fan” is usually a supporter of a particular team rather than the sport as a whole. Although some people support two clubs, one local and one from a top foreign league, supporters choose their favorite club based on various factors and develop loyalty to it (27). Nonetheless, along with supporting one team, individuals, who are more likely to be men than women, also define themselves as general fans of a sport (28). The typical behavior of the general fan consists of watching games or reading news about tournaments, teams, and players. However, it lacks the emotional connectedness or devotion to this activity, and this is due to a low level of attachment, considering there is no unique spatial or communal bonding. As highlighted by (29), sports media continues to evolve, enabling fans to engage more actively in immersive participation, essentially enhancing their involvement in augmented spectatorship. However, it remains evident that at its core, spectatorship remains a shared and prevalent activity. In contrast (30), introduces a distinct perspective on sports fandom, one that encompasses the integration of sports into everyday life, extending beyond traditional spectatorship. In doing so, he establishes the groundwork for the exploration of sports fandom as an ever-evolving and perhaps even fluid way of life. Moreover, (31) presents an analysis of sports enthusiasts using sociologist Anthony Giddens’ structuration theory. His findings suggest that the everyday practices of fans, referred to as “practical consciousness” (p. 293), are acquired and continually adapt through their social interactions. Essentially, the process of acquiring knowledge and embracing fan-related behaviors becomes an integral part of fans' daily lives. As the current article also underscores, these aspects play a vital role in shaping their collective identity.

In this article, I argue most previous definitions and behaviors still fall in the category of the traditional sports fan. However, the growing popularity of online sports fan communities has given rise to a new type of imagined community. The current conceptual article employs trends from Israeli sports, but it does not describe a phenomenon exclusive to this particular setting. In the global world, the diffusion of ideas and trends is shared instantly and virally. The common factors of the Israeli communities are humor and demonstration of knowledge, which are also an inseparable part of social human nature and the core characteristics of sports fandom. While the provincial-cultural viewpoint in Israeli society encouraged components of mediocrity traits as a part of these communities, this may be replaced in other countries by other relevant cultural characteristics. The present article seeks to lay the theoretical foundation for forming communities based on common social and cultural interests in the context of sports. This is to define the phenomenon in which fans of specific teams unite and become fans of the sport itself. This allows sports fans to devote more time to other fandom aspects, which provides them with many benefits. The emerging “fan of the sport” demands less commitment in everyday life, and it allows the individual to practice his fandom on a flexible schedule, has no passionate rivalries, does not emphasize sports success, and is generally less stressful. In other words, it may shift fandom to a more casual phenomenon, further away from the profound emotional connection and time-consuming lifestyle that has long been a part of sports fans' traditional identity.
